# Evidence to inform biopharmaceutical policy: call for research on the impact of public policies on investment in drug development

**DOI:** 10.1093/haschl/qxae129

**Published:** 2024-10-10

**Authors:** Sandra Barbosu, Kirsten Axelsen, Stephen Ezell

**Affiliations:** Information Technology and Innovation Foundation, Washington, DC 20001, United States; Tandon School of Engineering, New York University, Brooklyn, 11201, United States; DLA Piper, New York, NY 10020, United States; American Enterprise Institute, Washington, DC 20036, United States; Information Technology and Innovation Foundation, Washington, DC 20001, United States

**Keywords:** research and development for medicines, clinical study for medicines, investment in biopharmaceuticals, expected returns to innovation

## Abstract

This paper highlights the pressing need for updated, robust evidence to inform biopharmaceutical policy, particularly in light of recent initiatives such as the Inflation Reduction Act. Current estimates that inform such policies, including those from the Congressional Budget Office, rely on outdated data and models that fail to fully capture the complexities of modern investment decisions or the broader impact of policies on drug development in areas like oncology, rare diseases, and vaccines. Understanding how expectations of financial returns influence investment in all stages of drug development is critical for evaluating these policies’ long-term effects on innovation. This piece reviews the current evidence on the relationship between financial returns and research and development investment and considers how this evidence is being used to shape biopharmaceutical policy. It also highlights gaps in data and methodology, emphasizing the need for better models that reflect real-world trade-offs, investment risks, and therapeutic area-specific impacts. Finally, this paper calls for improved access to federal and private data to better inform evidence-based policymaking and to study policy impact on investments in the next generation of medicines, particularly in emerging fields like gene and cell therapies, where the implications of policy decisions are not yet fully understood.

## Introduction

Numerous studies have documented a positive, significant relationship between expected financial returns in the biopharmaceutical industry and investment in drug research and development (R&D).^1-4^ Recent work has also explored the impact of policies aimed to reduce drug prices, such as government price setting or the weakening of intellectual property protections, which lower financial returns and can disincentivize drug R&D. Ho and Pakes argue that current and proposed US price regulations, while benefitting US consumers in the short run, especially low-income and elderly populations, are likely to have harmful long-run effects by significantly reducing firms’ investment in highly welfare-improving R&D. This could have repercussions around the world, as pharmaceuticals, once developed, can benefit individuals in countries around the world.^5^ Philipson and Durie^6^ also show that US drug price setting provisions outlined in the Inflation Reduction Act (IRA) would lead to reduced R&D investment and a large decrease in new drugs developed, generating a large loss of life.

Yet the scope and magnitude of the trade-off between immediate savings from lower drug prices and future health benefits from clinical development remain poorly understood and quantified. There are few recent studies exploring this question, and estimates including those by the Congressional Budget Office (CBO) tend to rely on outdated or limited data that fail to capture the complexities of the current biopharmaceutical ecosystem—such as the mobility of capital or the full spectrum of investment decisions inherent in today's biopharmaceutical R&D ecosystem.^7^ Nevertheless, CBO estimates continue to inform policymakers’ decisions, although recent work suggests that those estimates may understate the negative impact of the IRA's price-setting provisions on future drug development.^6^

Congressional Budget Office has acknowledged the need for better data and more rigorous research to inform evidence-based policymaking and has called for new work on this topic.^8^ Building a robust evidence base is essential before implementing or extending significant policy changes to the healthcare system. This effort goes beyond federal research initiatives, since, historically, such research has originated in academic institutions and think tanks. To support rigorous evaluations and inform evidence-based policymaking, it is crucial to invest in this area through research grants and improved access to federal and private data on biopharmaceutical development costs, investments, and outcomes that better capture the complex R&D ecosystem.

The United States accounts for an estimated 40% of the global biopharmaceutical market, giving US policy considerable influence on expected financial returns for biopharmaceuticals.^9^ As such, changes in US policy could have significant negative consequences for global incentives for drug development. These changes could affect not only industry profits but also investment in academic research, clinical trials, and scientific methodologies, as well as human health and longevity worldwide.

## Challenges in understanding of the connection between expected returns and R&D investment in biopharma

Several notable studies have attempted to quantify the relationship between expected financial returns and R&D investment in the biopharmaceutical industry. Dubois et al.^1^ found that $2.5 billion in additional revenue is needed to invent 1 new chemical entity. Blume-Kohout and Sood^2^ found that the passage of Medicare Part D significantly increased R&D in therapeutic classes with higher Medicare market share. Acemoglu and Linn^3^ found that a 1% increase in potential market size in a therapeutic area, proxied by population aging, led to a 4%-6% increase in new drugs in that area. Finkelstein showed that a 1% rise in the utilization of existing vaccines, encouraged by public policies, boosted clinical trials for new vaccines by 2.5%-2.75%.^4^ These studies have contributed valuable insights, but more, and newer, work is needed for a comprehensive representation of how policy changes impact R&D investment and clinical development programs for novel therapeutics.

One challenge in estimating this relationship is that the required capital and the associated investment risks vary throughout the drug development lifecycle. Multiple entities, with different risk appetites and financial capabilities, are involved in different stages, but their risk tolerance and distinct investment strategies are not well documented. A model intended to capture the impact of policies on the biopharmaceutical industry should reflect the risk tolerance, access to capital, and alternative capital uses in each phase of drug development relative to the timing of the policy impact. Yet limited data and methods exist to shed light on how these decisions are made by different entities or why some drug candidates are not pursued even if expected returns are positive.^10^

Another challenge is identifying appropriate counterfactuals. For example, the implications of government price setting, a policy unprecedented before the IRA of 2022, are challenging to estimate due to the lack of analogs in markets of similar sizes to the US market. Recent efforts, including those undertaken by CBO, have focused on the effect of price setting on the number of new drugs developed in the future and have reported a wide range of estimates. While CBO found that 5 drugs would be lost by 2039 from current IRA price-setting provisions, Philipson and Durie estimate the effects to be 27 times larger, with 135 fewer new drugs by 2039, generating a loss of 331.5 million life years in the United States.^6,11^ Ho and Pakes^5^ find that proposals to extend price setting to all pharmaceuticals, while producing near-term benefits, would dramatically reduce firms’ investment in highly welfare-improving R&D for future generations of medicines. This would have global repercussions, as the benefits of a new drug, once developed, often extend around the world. Ho and Pakes argue for a more equitable distribution of the pharmaceutical R&D effort across high-income countries and examine the effects of setting a uniform international price for brand name drugs. They show that while aligning prices could cut US drug prices by more than half, it would lead to significant price increases in other nations, highlighting the complexity and wide-ranging consequences of global drug pricing dynamics on biopharmaceutical innovation.^5^

Existing research efforts may not fully reflect the impact of such policies on drug development, often failing to account for investment in clinical study with a distinct benefit in high-need populations, such as pediatric uses. Policies affecting expected financial returns from approved drugs impact new drug development and also R&D investment for existing drugs, including new indications. Current CBO estimates may not capture the broader impact on post-market development, competitor dynamics, and the entry of generics and biosimilars. The implications of government price setting on these dynamics—which have historically increased access to clinical data, broadened treatment options, and made drugs more affordable—have not been thoroughly studied.

## Limitations in CBO's current modeling of biopharmaceutical innovation

Congressional Budget Office uses various types of statistical and mathematical modeling to explore the impact of policy changes on biopharmaceutical innovation. For example, when assessing the effects of the IRA's price-setting provisions on drug development, CBO uses a combination of projection and decision-analytic models to estimate how price regulation might affect pharmaceutical innovation, costs, and savings and assess its outcomes on the economy and the healthcare system. Congressional Budget Office models have several limitations:

*Impact on post-market development*: The current focus on new drug development overlooks the implications of price setting for R&D for existing drugs later in the lifecycle. This investment is crucial for developing new indications; combination therapies, such as for cancer or HIV; and treatments for special populations such as children or individuals with comorbidities.

*Outdated estimates*: Current estimates of the relationship between expected financial returns and R&D investment rely on outdated data. The drug development landscape has evolved significantly, with more specialized drugs and complex development protocols, affecting both risks and costs.^12^ Updated data and modeling that better reflect the current complexities of biopharmaceutical investment would provide more relevant insights.

*Disparate impact on therapeutic areas and drug modalities*: Drug development has shifted significantly, with oncology and targeted therapeutics now comprising a much larger share of R&D investment compared with 2 decades ago. Existing data have not evaluated the effect on emerging modalities such as cell and gene therapies and RNA vaccines. This shift highlights the need for updated data and models that reflect today's drug development landscape and can evolve with the science of drug development.

*Variable risk tolerance in capital investment*: CBO modeling, based on limited data and a single representative firm, fails to account for varying risk profiles across firms. These models should better reflect capital mobility and diverse investment strategies throughout the drug development lifecycle. More comprehensive data, including insights from industry, such as quantitative firm data and qualitative research to better understand the risk profiles of different stakeholders, combined with modeling methodologies that account for heterogeneity across firms and investors, are needed to capture variations in risk tolerance, investment behavior, and decision-making, providing a more realistic representation of industry dynamics.

*Effect on competitor drugs*: Government price setting may reduce the revenue of competitor drugs in the same therapeutic area, leading to lower prices and a drop in R&D and clinical development within that therapeutic area. This effect, akin to the dynamics of generic drug entry, remains uncertain and requires further investigation.

*Competitive market for generics and biosimilars*: Updated evidence is needed to assess the impact of government price setting on branded drugs on the entry and pricing of generics and biosimilars in Medicare.

*Lack of focus on health or equity*: Current CBO models emphasize, often overlook, the broader implications of policies on health outcomes and health equity, such as the potential to restrict patient access to innovative treatments or to exacerbate health disparities.

Expanding the evidence base is crucial for evaluating policy impacts in the biopharmaceutical industry. Studies should be transparent, replicable, reproducible, and independently verified. This is especially important considering the range of methodologies and estimates of current studies. To improve evidence-based policymaking, novel research should address existing limitations and incorporate insights from academic and industry experts on important topics such as the effects of policies on new drug development, access to medicines for diverse populations—including those often underserved by the healthcare system—and health outcomes. Enhancing access to federal data, including comprehensive records from the Food and Drug Administration, such as clinical trials and drug and device applications and approvals, would be crucial to support such studies. Policymakers must call for more precise and comprehensive evidence to better understand the implications of significant policy changes prior to implementing them. Moreover, to support the expansion of this evidence base, federal funders such as the National Institutes of Health should be supported to open dedicated funding lines in this area.

## Call for papers: ITIF research grants

Congressional Budget Office plays a critical role in the legislative process by providing independent, nonpartisan economic, and budgetary analysis for proposed laws. Improved CBO modeling offers policymakers more precise insights into policy impacts, reducing the risk of unintended effects. This can help tailor policies to achieve specific goals, such as fostering biopharmaceutical innovation and improving public health. More robust analysis also helps in weighing the costs and benefits of new policies, ensuring decisions are grounded in solid evidence to produce more effective outcomes.

Several open research questions on the topic of biopharmaceutical policy and R&D investment could add valuable insights to the current evidence base. They are summarized in Table 1.

**Table 1.** Open research questions.

Research topic	Open questions
Quantify trade-offs	What is the elasticity of R&D investment with respect to changes in expected financial returns? How does this elasticity vary across therapeutic areas and drug modalities?
Innovation impact	How do government policies, such as price setting, influence the types of drugs developed (radical vs incremental innovation)? What is the long-term impact on breakthrough therapies for rare diseases?
Global implications	How do US policy changes impact global drug development, given the US is the single largest global biopharmaceutical market? What are the spillover effects on global pharmaceutical innovation and access?
R&D prioritization	How do changes in expected financial returns affect prioritization across different therapeutic areas or stages of drug development?
Economic and health outcomes	What are the implications for health equity, such as access to new therapeutics?
Risk	How do government policies, such as price setting, affect the level of risk of different stakeholders (pharmaceutical companies, venture capitalists, and public funding bodies)? How do these different stakeholders respond to changes in expected returns?
Post-market developments	How do policies like price setting impact post-market developments such as new indications, combination therapies, and pediatric formulations?
Comparative policy analysis	How do different price setting mechanisms (direct price controls, reference pricing, and value-based pricing) compare in their impact on R&D incentives?
Data and methodology	What are examples of appropriate methodologies for studying the impact of policy changes on R&D investment? How can access to diverse, granular, high-quality data be expanded, and what role do emerging privacy-enhancing technologies (eg, federated learning and fully homomorphic encryption) play in enabling access?
Measurement	What types of dependent variables capture innovation in the biopharmaceutical industry (eg, number of new drugs, clinical trials, trials with new therapeutic targets, clinical improvements, patents, and high-impact publications)?
Interdisciplinary approaches	How can insights from fields such as economics, management, health policy, innovation, and public health be integrated to provide a more comprehensive understanding of the impacts of biopharmaceutical policies?

Abbreviation: R&D, research and development.

Building a more robust evidence base on topics outlined in Table 1 and beyond could provide a deeper understanding relevant to policymakers around the world of how financial returns affect future drug innovation across therapeutic areas and drug modalities. Insights into the factors that guide R&D investment decisions help shape international collaborations, public funding priorities, and regulatory frameworks that encourage innovation in the medical areas of greatest need. As the largest global biopharmaceutical market, policy changes in the United States can have far-reaching implications, affecting drug pricing, access, and innovation across borders. The spillover effects on global innovation and access to medicines can inform collaborative efforts to address global health challenges. As Ho and Pakes describe,^5^ pharmaceuticals are global goods, which, once developed, can benefit countries around the world. Therefore, policies that impact US drug development can have significant global repercussions and can help inform policymakers in other countries, emphasizing the relevance to policy approaches around the world.

To encourage further research in this area, the Information Innovation and Technology Foundation (ITIF), an independent nonprofit, nonpartisan research and educational institute for science and technology policy, is offering research grants for new work. These grants are available to individuals in academic institutions or private organizations working on the themes outlined in this commentary. Eligible researchers are encouraged to apply and to share this opportunity broadly.

## Supplementary Material

qxae129_Supplementary_Data

## Data Availability

Data used in this commentary are cited, no additional data availability.
